# Discussion of a Simple Method to Generate Descriptive Images Using Predictive ResNet Model Weights and Feature Maps for Recurrent Cervix Cancer

**DOI:** 10.3390/tomography11030038

**Published:** 2025-03-20

**Authors:** Destie Provenzano, Jeffrey Wang, Sharad Goyal, Yuan James Rao

**Affiliations:** 1 School of Engineering and Applied Science, George Washington University, Washington, DC 20052, USA; dprovenzano23@gwu.edu; 2 Department of Radiation Oncology, School of Medicine and Health Sciences, George Washington University, Washington, DC 20052, USA; jywang@gwmail.gwu.edu (J.W.); shgoyal@mfa.gwu.edu (S.G.)

**Keywords:** cervix cancer, ResNet, deep learning, XAI, machine learning, model explainability, generated images, most important feature maps, radiotherapy, radiation therapy

## Abstract

Background: Predictive models like Residual Neural Networks (ResNets) can use Magnetic Resonance Imaging (MRI) data to identify cervix tumors likely to recur after radiotherapy (RT) with high accuracy. However, there persists a lack of insight into model selections (explainability). In this study, we explored whether model features could be used to generate simulated images as a method of model explainability. Methods: T2W MRI data were collected for twenty-seven women with cervix cancer who received RT from the TCGA-CESC database. Simulated images were generated as follows: [A] a ResNet model was trained to identify recurrent cervix cancer; [B] a model was evaluated on T2W MRI data for subjects to obtain corresponding feature maps; [C] most important feature maps were determined for each image; [D] feature maps were combined across all images to generate a simulated image; [E] the final image was reviewed by a radiation oncologist and an initial algorithm to identify the likelihood of recurrence. Results: Predictive feature maps from the ResNet model (93% accuracy) were used to generate simulated images. Simulated images passed through the model were identified as recurrent and non-recurrent cervix tumors after radiotherapy. A radiation oncologist identified the simulated images as cervix tumors with characteristics of aggressive Cervical Cancer. These images also contained multiple MRI features not considered clinically relevant. Conclusion: This simple method was able to generate simulated MRI data that mimicked recurrent and non-recurrent cervix cancer tumor images. These generated images could be useful for evaluating the explainability of predictive models and to assist radiologists with the identification of features likely to predict disease course.

## 1. Introduction

Despite advances in treatment and screening for tumors of the cervix, Cervical Cancer remains a major burden worldwide and is the fourth most common cancer affecting women [1]. Cervical Cancer treatment, through methodologies such as radiation therapy (RT), is increasingly more effective when tumors are caught early, with a 95% cure rate for early-stage treatments; however, this rate decreases to 40–60% for locally advanced disease [2,3]. Recent research has seen groundbreaking advancements in other alternative treatments for Cervical Cancer as well [4,5,6]. Cervical Cancer recurrence is defined as the local, regional, or distant growth of a tumor at least 6 months after the regression of the primary tumor [7]. MRI has been highly effective at the detection of recurrent cervix cancer after primary treatment [8]. This makes screening for early-stage disease and later recurrence through imaging methods such as Magnetic Resonance Imaging (MRI) of utmost importance.

Recent years have seen the use of Machine Learning (ML) grow in popularity as a potential method to distinguish malignant cervix tumors from MRI data [9]. Convolutional Neural Networks (CNNs) are a form of ML known for their simplicity and high accuracy that utilize a convolutional layer to automatically select features as model inputs. Convolutional Neural Networks (CNNs) have previously been shown to be incredibly effective at distinguishing malignant cervix tumors on MRI [10,11,12,13]. We also previously demonstrated that a type of CNN called a Residual Neural Network (ResNet) can identify cervix tumors likely to experience recurrence after radiotherapy treatment from MRI data with high accuracy [14]. But despite the plethora of highly accurate CNNs available, the adoption of ML models for clinical practice still faces many challenges [15,16]. One large contributor to this lack of adoption is the lack of inherent explainability present in models like CNNs that utilize automatic feature detection methods [17]. Many methods of explainable AI (XAI) or model explainability exist to attempt to explain model selections like GradCAM, SHAP, and LIME [18,19,20]. But studies have shown that these methods do not adequately explain model selections and are not sufficient for potential models to be used in clinical practice [21,22]. For medical imaging studies, GradCAM is often the gold standard for explainability; it seeks to produce a qualitative heatmap representation of the model selections, and has been used to explain model selections for brain tumors [23], or Parkinson’s Disease [24]. SHAP and LIME have been used for many use cases such as prediction of brain stroke from CT using vision transformers [25]. Techniques such as GradCAM are typically considered “attribution based” and use gradient (GradCAM) or perturbation based (SHAP, LIME) techniques to attempt to explain model selections [26]. Other explainability techniques do exist, such as attention networks, feature analytic methods, and the generation of simulated images using techniques such as GANs [27,28,29]. The use of simulated images through GANs has been explored for use cases, such as a study evaluating model selections and explainability for chest radiographs, but were found to be limited by computational restrictions [30]. This study proposed a method to overcome these computational limitations by generating simulated images using the most important feature maps.

Our previous study explored the use of the most important feature map as a method to create a quantitative explainability metric [31]. We also demonstrated the ability to use a combination of the most important feature maps for images run through a model for recurrent cervix cancer to generate a simulated image representative of the model selections [32]. In this study, we detail the exact methodology to generate simulated images for a dataset from a highly accurate predictive model for recurrent cervix cancer and discuss the resulting generated simulated MRI image.

## 2. Materials and Methods

### 2.1. Data Collection

Data were collected from The Cancer Genome Atlas Cervical Squamous Cell Carcinoma and Endocervical Adenocarcinoma Collection (TCGA-CESC) cohorts available on the Cancer Imaging Archive (TCIA) [33,34]. T2-Weighted (T2W) MRI data for 28 women with cervix cancer who had received radiotherapy treatment was identified from the 54 subjects in the TCGA-CESC collection for use in the study. Clinical data for the dataset was evaluated to identify 7 women with recurrent metastases after treatment.

All available DICOM image slices for the data were collected resulting in 3-Dimensional initial images for model training process. Final feature map generation was performed on the entire 3-D dataset to output the highest weighted feature map, resulting in a final 2-D image.

### 2.2. Simulated Images Generated by Custom Algorithm

First, a Residual Neural Network (ResNet) was identified as the ideal model of choice for training purposes due to its simplicity, speed, and high performance on small medical imaging datasets. Multiple initial model frameworks were tested to arrive at ResNet (including Densenet, VGG, Inception, etc.). For this problem, ResNet provided the best results the quickest and was chosen as the optimal framework. Pre-built ResNet framework was used from the Tensorflow (2.19) python package to train initial model [35]. Due to the small nature of the dataset used in this experiment, a hybrid model approach was selected that used a transfer learning process combined with additional added model layers to provide a way to generate the most potential specialized features for these data. This model used a standard training/testing process with 5-fold cross validation (80/20 training/testing split by default) to optimize the use of the small dataset and still provide statistical significance. A shuffle test was employed to ensure no randomly shuffled run could achieve the same accuracy. The tf.keras.utils package within python Tensorflow was used to load the dataset from a directory and provide default tensorflow preprocessing needed to organize data and ensure images were prepared for model run. No additional preprocessing to MRI data were employed besides Tensorflow defaults. Transfer learning, or the process of removing the final prediction layer from a pre-trained model to use features generated from a larger dataset to train a model on a new dataset, was used for initial training process on 3D T2W MRI data. We previously observed that the transfer learning process generates non-specific features that can encompass the entire image regardless of feature importance to the new task [31]. As such the entire 3-D MRI image and all available data for each patient was used to ideally force the model to identify the most important DICOM slice for feature map generation and hone in on the cervix tumor itself. Additional dense layers were added to ResNet model to also further hone in on features specific to the Cervix Cancer dataset. Model was trained on patients separated into recurrent or non recurrent pre-treatment imaging MRI data and passed through 5-fold cross validation and a shuffle test for statistical significance. Final highly accurate model of 93% accuracy was used for feature map generation. Model was trained through a standard 80%/20% training/testing split with 5-fold cross validation and a shuffle test as detailed below.

Next, the model was tested on the TCGA-CESC image dataset consisting of the 3D T2W MRI data for patients with and without recurrent cervix cancer tumors to obtain corresponding feature maps and model weights for each region of the MR images. These feature maps can be generated for any layer within a model passed through Tensorflow by selecting the relevant layer and passing it through the model.predict function. For this experiment, the final model layer before the predictive score generation was used to generate feature maps. This resulted in thousands of potential feature maps from the ResNet for each MR image for a patient. The term feature map used here refers to the feature maps generated by the ResNet model. Typically, feature maps are thought to contain one “feature” per map, but a feature here does not necessarily mean one pixel or region of the image. Due to the convolutional layer and filtering process that a CNN employs, a feature can often consist of multiple parts of the image that are ultimately assigned excitatory or inhibitory weights for that map. Lower/earlier layers of a CNN like a ResNet can output features that sometimes even resemble highlighted portions of the original image, but the later layers can often seem more abstract. For medical images like MRI data where the features can seem abstract without highly specialized medical training, these feature maps may seem particularly abstract. In a typical model selection process, these features are then fed to one final layer that outputs a probabilistic score to summarize the weights of these features.

Next, the feature map with the corresponding highest weights attributed to it by the model was identified as the most important feature map for each individual image. Only one feature map was selected as the most important feature map. The use of the 3-D T2W MR image for training and selection of one 2-D T2W MR image for feature map generation was performed to hopefully hone in on the DICOM slice with the cervix cancer tumor for each patient.

Finally, these most important predictive features from each most important feature map were then stacked for the entire dataset to create one new simulated image summarizing the data.

**Figure 1:**
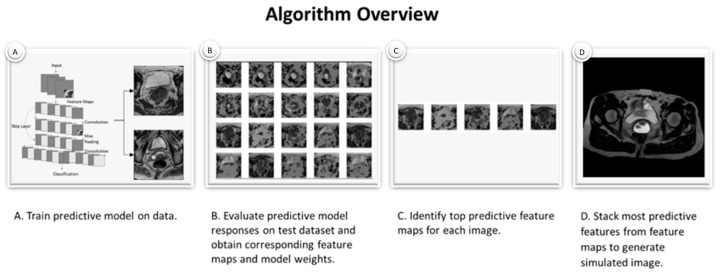
Overview of algorithm workflow to generate simulated data.

The stacking process used for this final simulated image utilizes a standard generative model procedure (Figure 1) [36]. For this we define a joint probability distribution P(X,Y) where P(X) is the latent space created by the various feature maps from the model selection process where each feature map contains a matrix (m × n) of the model weights, and P(Y) is the randomly initialized matrix (m × n) where outcomes are to be mapped. The m x n is defined by the initial image size and model input. Observations (x) from the latent space are mapped to y such that P(X) is maximized. This approach was popularized for deep learning use cases through GANs [37]. However, unlike GANs, which seek to minimize P(X) and maximize P(Y) by repeatedly pulling samples (x) from P(X), this approach utilizes only one feature map from each image and thus can initialize P(Y) to be 0 and pull the x such to maximize P(X) from the latent space. On a dataset with more samples special care should be taken as to consider if P(X) should simply be maximized, or if an average, random, or other strategy should be undertaken to map values to P(Y).

### 2.3. Image Classification

Final generated simulated image was passed back through initial ResNet algorithm using standard model processing procedure for prediction score to identify likelihood of recurrence.

### 2.4. Statistical Significance

Initial model was tested for statistical significance through 5-fold Cross Validation where the data are separated into 5 separate 80/20 training/testing splits. This creates 5 datasets where 80% of the data are in the training set and a separate 20% testing set from each of the other 5 datasets is used for testing the data. This way the model is trained on 100% of the data and tested on 100% of the data to amplify the ability of a small dataset to produce statistically significance results.

A shuffle test was used to validate that the model accuracy could not be achieved on any randomly shuffled run. To perform this test, the labels were randomly shuffled 100 times and the model was retrained on each of the shuffled labels to see if any random run could achieve the initial accuracy. As none did this resulted in a *p* < 0.01.

## 3. Results

### 3.1. Model Accuracy and Sample Feature Maps

**Figure 2:**
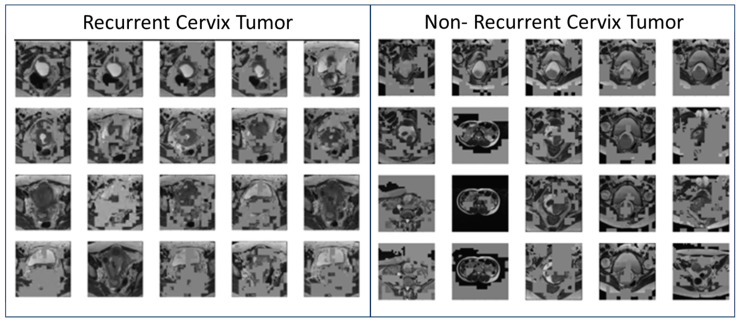
Sample Feature Maps from predictive Residual Neural Network (ResNet) Trained on T2-Weighted (T2W) Magnetic Resonance Imaging (MRI) data to identify recurrent vs. non-recurrent cervix cancer tumors for women with cervix cancer who had undergone radiotherapy treatment.

Initial model for recurrent cervix cancer after radiotherapy treatment was able to achieve an accuracy of 93% at prediction of tumors likely to recur. Feature maps were generated for each individual image by passing each image in the dataset through the model and outputting the last convolutional layer before the final prediction function. Sample feature maps for recurrent cervix tumors and non-recurrent cervix tumors are displayed in Figure 2.

### 3.2. Generated Simulated Cervix Tumor Images

**Figure 3:**
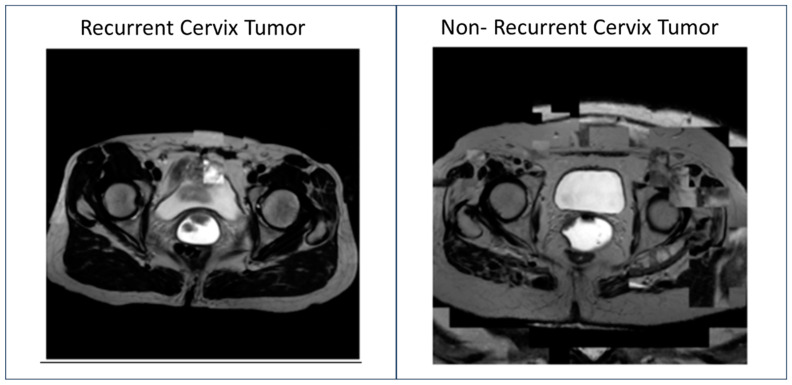
Generated Simulated MRI Images from predictive Residual Neural Network (ResNet) trained on T2-Weighted (T2W) Magnetic Resonance Imaging (MRI) data to identify recurrent vs. non-recurrent cervix cancer tumors for women with cervix cancer who had undergone radiotherapy treatment.

Most important (highest weighted) feature maps from the cohort of patients with recurrent cervix tumors and non-recurrent cervix tumors from each image were then stacked to create two simulated images (Figure 3). Due to the small sample size, these feature maps were able to be stacked simply by taking the feature with the highest weight and assigning the new image pixel the pixels at that weight. Model was able to predict the generated recurrent cervix tumor image as recurrent and the generated non-recurrent cervix tumor image as a non-recurrent cervix tumor. Radiation oncologist identified the two generated images as having characteristics consistent with a cervix MRI.

## 4. Discussion

Better methods of model explainability (XAI) are urgently needed to increase adoption of machine learning models within clinical medicine. This study demonstrated a potential new qualitative method that could highlight the most important feature maps for a predictive model for cervix cancer. This experiment used a small dataset of 28 (21 Non-Recurrent, 7 Recurrent) to produce two generated simulated images representative of a combination of the ResNet model most important feature maps. These images were able to be identified by both a clinician (Radiation Oncologist) and the predictive model as cervix tumor images. These images also provided a summary image of the most important features a model would select for an entire dataset. By combining the features in this manner, the image was able to showcase the regions a model may find important from all the images, including those potentially not clinically relevant.

The image containing a recurrent cervix tumor showcased a very large mass consistent with a combination of features attributed to different cervix tumors on the seven recurrent images. But the presence of features corresponding to regions of the cervix outside the cervix tumor on these final simulated images indicated that these models were also using information besides that of the cervix tumor itself to make its predictions. The use of transfer learning, which uses generated features from a different model to make new predictions on a small dataset, was one reason for the presence of these exterior features. It can be assumed that a dataset trained on ImageNet for various other classes (car, cat, dog, etc.) would seek to identify features identifying these classes initially, so it follows that a transfer learning process will almost always identify some features that are not clinically relevant. Additionally, certain features about the cervix and surrounding tissue, such as parametrial invasion, vaginal invasion, pelvic side wall invasion, bladder or rectum invasion, or general enlargement of the cervix, can indicate that a severe tumor is more likely to recur. The features on the exterior regions of the pelvis with the exception of lymph node involvement and additional metastases are not as likely to be predictive of recurrence. These features highlighting the exterior regions of the pelvis also raise questions regarding what is required for a model to be clinically relevant.

The qualitative nature of these generated simulated images does lead to some limitations. For one it requires a user to observe the images and make conclusions, which can introduce additional bias Additionally, it does not provide a quantitative way of evaluating clinical relevance. This is a common problem with many explainability techniques beyond attempts to explain model selections through simulated images, such as the attribution based methods like GradCAM. Many studies have explored the limitations this can impose on interpretability of these methods and how to introduce quantitative measures, as it can lead the observer to see what they want to see rather than what is there [38]. Interestingly enough, the specialized nature of many of these popular perturbation and attribution based methods has led to essentially a research field in itself where researchers attempt to explain the explanations. Although outside of the scope of this study, one method our previous study explored to create a quantitative metric out of current model explainability techniques was to evaluate if a feature map co-localized with an important clinical region, and it would be interesting to see in future work if these simulated images contain co-localized regions as well [31]. This study was also limited in that it used a small dataset. More data are urgently needed to fully evaluate this methodology and future work should explore this on larger datasets with different types of image classification problems including different cancer types, medical imaging tasks, and even other tasks entirely. As this study took the highest weighted features from a small dataset, the individual features on the final image were able to be simply stacked. However, additional calculations may be needed to determine what features to display on the final image if more data were to be used. Future work would benefit from testing this methodology on more data and identifying relevant calculations needed where important features from multiple images overlap. These generated simulated images could be useful for evaluating the explainability of future predictive models and to assist radiologists with identification of features likely to predict disease course for an input dataset. It should be noted that many systems of XAI for medical imaging has so far also been focused on qualitative representations [39]. Future work using quantitative measures in combination with techniques such as this one would greatly benefit both the clinician and model developer to creating implementable AI.

The methodology in this study was limited to a small dataset and cancer population, however, could be generalized to other cancers, medical images, or image classification tasks. More data are urgently needed to validate these results and the conclusions here represent that of a pilot study on limited data. Although this methodology does not seek to be the final or only method to explain a model’s selections, it does seek to provide better qualitative insight into visualization of the feature maps used. It also does so for an entire dataset condensed to one image, making it a unique contribution to the field Other explainability techniques such as Grad-CAM, SHAP, or LIME work on individual images but often do not show a global summary of model selections for a dataset, so in that way this small method hopes to contribute a potential way to change this. Explainable techniques, in general, are urgently needed for the implementation of models in clinical medicine. Without XAI, inadequate or nonrepresentative datasets, inadequate training, or simple probabilistic representations of the data that cannot accurately determine true or false can lead to systematic biases in model outputs that raise legal and ethical concerns regarding if these models can be used at all [40,41]. Adding qualitative representations such as that depicted here could assist a modeler and clinician to identify if the features used to make model predictions are looking in the correct region or if they can function without biases on new data. Cost-effectiveness of AI methods should also be taken into account in addition to legal, ethical, and explainable concerns; studies have explored this but more work is urgently needed to ensure these models can be implemented in clinical practice [42].

## 5. Conclusions

This study detailed the results of a potential methodology to generate simulated MRI data that mimic recurrent and non-recurrent cervix cancer tumor images. The images generated by this experiment were found to be consistent with recurrent and non-recurrent cervix cancer by a radiation oncologist and via model prediction. The features present on these images indicated that the model was considering data on the entire pelvis as important, raising questions about the need for model features to be clinically relevant. Further refinement of this methodology is needed for use on broader datasets with more data.

## Data Availability

All data are available from the TCGA-CESC database on the TCIA website. https://www.cancerimagingarchive.net/collection/tcga-cesc/. (accessed on 1 August 2024).

## Abbreviations

The following abbreviations are used in this manuscript:

RT	Radiation Therapy
ResNet	Residual Neural Network
ML	Machine Learning
AI	Artificial Intelligence
XAI	Explainable AI
MRI	Magnetic Resonance Imaging
